# Self-reported insomnia as a marker for anxiety and depression among migraineurs: a population-based cross-sectional study

**DOI:** 10.1038/s41598-019-55928-8

**Published:** 2019-12-20

**Authors:** Kyung Min Kim, Dong Hyun Lee, Eun Ju Lee, Yun Ho Roh, Won-Joo Kim, Soo-Jin Cho, Kwang Ik Yang, Chang-Ho Yun, Min Kyung Chu

**Affiliations:** 1 Department of Neurology, Severance Hospital, Yonsei University College of Medicine, Seoul, Korea; 2 Biostatistics Collaboration Unit, Department of Biomedical Systems Informatics, Yonsei University College of Medicine, Seoul, Korea; 3 Department of Neurology, Gangnam Severance Hospital, Yonsei University College of Medicine, Seoul, Korea; 4 Department of Neurology, Dongtan Sacred Heart Hospital, Hallym University College of Medicine, Hwaseong, Korea; 5 Sleep Disorders Center, Department of Neurology, Soonchunhyang University College of Medicine, Cheonan Hospital, Cheonan, Korea; 6 Department of Neurology, Bundang Clinical Neuroscience Institute, Seoul National University Bundang Hospital, Seongnam, Korea

**Keywords:** Anxiety, Depression, Migraine, Sleep disorders

## Abstract

Anxiety, depression, and insomnia are highly prevalent among migraineurs and are associated with negative health consequences. Anxiety and depression, however, unlike insomnia, are usually underdiagnosed, due to less self-reporting of these two conditions. The aim of the present study was to evaluate the risk of anxiety and depression in migraineurs with self-reported insomnia, using a general population-based sample. We used data from a nationwide population-based survey on headache and sleep, the Korean Headache-Sleep Study. Of all 2,695 participants, 143 (5.3%), 268 (10.0%), 116 (4.3%), and 290 (10.8%) were classified as having migraine, anxiety, depression, and self-reported insomnia, respectively. The risk of anxiety (odds ratio [OR] = 7.0, 95% confidence interval [CI] = 3.0–16.7) and depression (OR = 3.3, 95% CI = 1.3–8.5) was significantly increased in migraineurs with self-reported insomnia. The sensitivity, specificity, positive predictive value (PPV), and negative predictive value (NPV) for anxiety in migraineurs with self-reported insomnia were 46.5%, 89.0%, 64.5%, and 79.5%, respectively. For depression, the sensitivity, specificity, PPV, and NPV were 41.7%, 82.4%, 32.3%, and 87.5%, respectively. Self-reported insomnia is likely to be comorbid with anxiety and depression in migraineurs and could thus be a useful predictor of anxiety and depression in migraine.

## Introduction

Anxiety and depression are common comorbidities of migraine and are associated with an exacerbation of migraine symptoms^1,2^. They are also risk factors for transformation to chronic migraine (CM), a chronic disabling subtype of migraine that is usually refractory to treatment^3,4^. The accurate diagnosis and proper treatment of anxiety and depression are thus important in the management of migraine.

Insomnia is another condition frequently encountered in migraineurs^5–9^, likewise associated with an exacerbation of migraine symptoms and a risk factor for transformation to CM^10^. Similar to migraine, insomnia shows a significant association with anxiety and depression^11^.

Anxiety and depression are typically underdiagnosed and undertreated^12,13^. Clinicians are often unaware of the presence of anxiety and depression when caring for migraine patients^14^, and, as a result, migraineurs are often not optimally treated. Symptoms of insomnia, in contrast, are commonly reported, and the condition is easily recognized^15^.

Insomnia can be assessed using various methods, and self-reporting has proved useful^15,16^. We hypothesized that a patient’s risk for anxiety and depression significantly increases when he/she self-reports insomnia, and self-reported insomnia could thus be a useful marker for anxiety and depression in migraineurs. The aim of the present study was to investigate the diagnostic value of self-reported insomnia for anxiety and depression among migraineurs, using data from a population-based sample.

## Methods

### Sampling methods and survey process

We used data from the Korean Headache-Sleep Study (KHSS) that was conducted from November 2011 to January 2012. The KHSS represents a nationwide, population-based, cross-sectional survey on headache and sleep in Korea. It covers the entire country with the exception of Jeju-do and uses a two-stage clustered random sampling method based on population and sociodemographic distributions. Trained interviewers conducted face-to-face interviews using a structured questionnaire that included items regarding symptoms of insomnia, anxiety, depression, and headache. We informed participants that our survey them was a social health issue than migraine, insomnia and mood problems during recruitment to prevent interest bias. The survey was conducted via a structured interview using questionnaire. Individuals who previously participated in a survey on headache, sleep and mood problems were excluded. Our survey was conducted from November 2011 to January 2012. The detailed process of the survey has been described previously^17^. Written informed consent was obtained from all participants during the interview. The KHSS was approved by the Institutional Review Board/Ethics Committee of Hallym University Sacred Heart Hospital (IRB No. 2011-I077). All methods were performed in accordance with the relevant guidelines and regulations.

### Assessment of migraine

The headache profile included in the questionnaire was based on the classification in the second edition of the International Classification of Headache Disorders (ICHD-II)^18^. The migraine diagnosis was based on the ICHD-II for migraine without aura (code 1.1). We did not investigate the presence of an aura, because it is difficult to identify aura symptoms in a population-based survey^19^. Therefore, individuals with migraine in this study comprised both those with an aura (code 1.2) and those without an aura. The validity of the migraine diagnosis was ensured by comparing the questionnaire results with a doctors’ diagnosis during an additional telephone interview. The sensitivity and specificity of the questionnaire for the diagnosis of migraine were 75.0% and 88.2%, respectively^20^.

### Assessment of self-reported insomnia and insomnia symptom

Self-reported insomnia was defined as the participant answering “yes” to the following question: “Do you have insomnia?”

We evaluated the presence of insomnia symptoms by using the Insomnia Severity Index (ISI). Participants with ISI scores of 10 or higher were defined as having insomnia symptom^21,22^. Among those who satisfy this definition (ISI score ≥ 10), we further classified the subtypes of insomnia symptoms as difficulty in initiating sleep (DIS), difficulty in maintaining sleep (DMS) and early morning awakening (EMA) if a participant responded with ≥2 on the scale (intermediate or higher) for those items.

### Assessment of anxiety and depression

We used the Goldberg Anxiety Scale (GAS) for the assessment of anxiety. The GAS is composed of nine items: four screening items and five supplementary questions. A participant who gives a positive response to two or more screening questions and five or more questions overall is considered to have anxiety. The Korean version of the GAS has been evaluated and has shown a sensitivity of 82.0% and a specificity of 94.4%^23^.

The Patient Health Questionnaire (PHQ)-9 was used to diagnose and assess the severity of depression^24^. The PHQ-9 is a nine-item questionnaire based on the 4th Edition Diagnostic and Statistical Manual of Mental Disorders criteria of depression. Participants with PHQ-9 scores of 10 or more were defined as having depression. The Korean version of the PHQ-9 has been evaluated and has shown a sensitivity of 81.1% and a specificity of 89.9%^25^.

### Statistical analyses

Data normality was evaluated using the Kolmogorov-Smirnov test. After ensuring a normal distribution, Student’s t-tests were conducted for analyzing differences between continuous variables. For comparing categorical variables, Chi-square tests were used. The Statistical Package for Social Sciences version 23.0 (SPSS 23.0; IBM, Armonk, NY, USA) was used for all statistical analyses except for comparisons of sensitivity, specificity, positive predictive value (PPV) and negative predictive value (NPV). Accuracy expressed as the proportion of correctly classified subjects (true positive and true negative) among all subjects. Odds ratio (OR) is defined by the ratio of the odds of having anxiety or depression in a selected group to the odds of having anxiety or depression in an unselected group. These values were compared using Chi-square tests for two independent groups (migraineurs vs. non-migraineurs) and generalized estimating equation methods for two dependent groups (self-reports vs. ISI scores ≥10 among migraineurs) using SAS version 9.4 (SAS Inc., Cary, NC, USA). Receiver operating characteristic (ROC) curves and the area under the curve (AUC) for each ROC were obtained to measure the discrimination capacity of anxiety and depression. Statistical significance was set at *p* < 0.05 two-tailed.

## Results

### Survey

**Figure 1:**
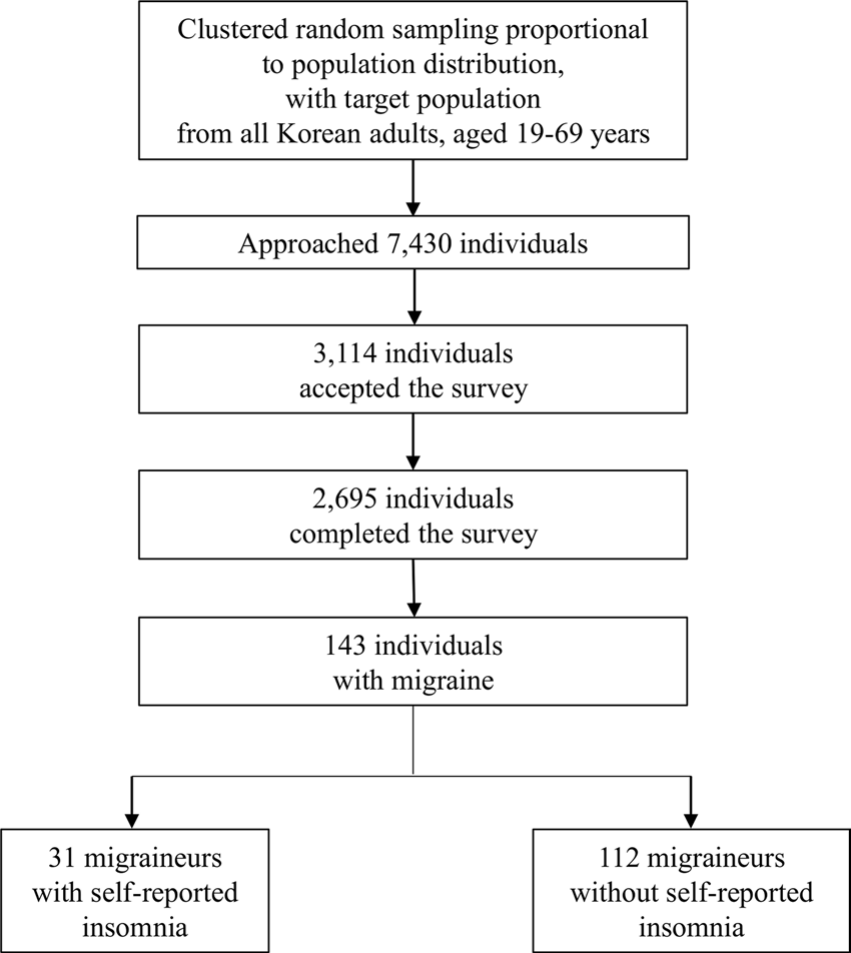
Survey participants of the Korean Headache-Sleep Study.

Clustered random sampling was conducted, proportional to population distribution, and 7,430 individuals were contacted by the interviewers. Among them, 3,114 individuals initially agreed to participate in the survey, but 419 later declined to participate. A total of 2,695 individuals thus completed the survey and were included in the analyses (Fig. 1). The survey participants did not significantly differ from the general Korean population in demographic characteristics, such as sex, age, size of residential area, and education level (Table 1).

**Table 1:** Demographic characteristics of the survey participants, total population, and participants with migraine, anxiety, depression, and self-reported insomnia.

	Survey participants N (%)	Total population N (%)	p-value	Migraine N, % (95% CI)	Anxiety N, % (95% CI)	Depression N, % (95% CI)	Self-reported insomnia N, % (95% CI)
**Sex**							
Men	1,345 (49.3)	17,584,365 (50.6)	0.854	36, 2.7 (1.8–3.5)	109, 8.1 (6.6–9.6)	43, 3.2 (2.3–4.2)	85, 6.3 (5.1–7.6)
Women	1,350 (50.7)	17,198,350 (49.4)		107, 7.9 (6.5–9.4)	159, 11.8 (10.1–13.5)	73, 5.4 (4.2–6.6)	168, 12.4 (10.7–14.2)
**Age**							
19–29	542 (20.5)	7,717,947 (22.2)	0.917	25, 4.5 (2.7–6.2)	53, 9.6 (7.2–12.1)	23, 4.1 (2.5–5.8)	40, 7.4 (5.2–9.6)
30–39	604 (21.9)	8,349,487 (24.0)		42, 7.0 (4.9–9.1)	51, 8.7 (6.4–11.0)	32, 5.4 (4.6–7.3)	41, 6.8 (4.8–8.8)
40–49	611 (23.1)	8,613,110 (24.8)		39, 6.5 (4.5–8.4)	67, 11.0 (8.5–13.5)	24, 4.0 (2.5–5.5)	56, 9.2 (9.6–11.5)
50–59	529 (18.9)	6,167,505 (17.7)		22, 4.1 (2.4–5.9)	53, 9.9 (7.3–12.5)	22, 4.2 (2.5–6.0)	59, 11.1 (8.5–13.8)
60–69	409 (15.6)	3,934,666 (11.3)		15, 3.9 (2.0–5.7)	14, 10.8 (7.8–13.8)	15, 3.7 (2.0–5.5)	57, 13.9 (10.6–17.3)
**Size of residential area**							
Large city	1,248 (46.3)	16,776,771 (48.2)	0.921	76, 6.1 (4.8–7.5)	130, 10.4 (8.7–12.1)	57, 4.6 (3.4–5.7)	115, 9.2 (7.6–10.8)
Medium-to-small city	1186 (44.0)	15,164,345 (43.6)		48, 4.0 (2.9–5.2)	112, 9.5 (7.8–11.2)	47, 4.0 (2.9–5.1)	107, 9.0 (7.4–1.07)
Rural area	261 (9.7)	2,841,599 (8.2)		19, 7.4 (4.2–10.6)	26, 10.0 (6.3–13.6)	12, 4.7 (2.1–7.3)	31, 11.9 (7.9–15.8)
**Education level**							
Middle school or less	393 (14.9)	6,608,716 (19.0)	0.752	22, 5.5 (4.2–7.7)	55, 13.9 (10.5–17.4)	20, 5.2 (3.0–7.4)	72, 18.3 (14.5–22.2)
High school	1,208 (44.5)	15,234,829 (43.8)		60, 5.0 (3.8–6.3)	111, 9.2 (7.5–10.8)	49, 4.1 (3.0–5.2)	106, 8.8 (7.2–10.4)
College or more	1,068 (39.6)	12,939,170 (37.2)		60, 5.6 (4.3–7.0)	100, 9.5 (7.7–11.2)	47, 4.4 (3.2–5.7)	73, 6.8 (5.3–8.4)
Not responded	26 (9.6)			1, 3.8 (0.0–11.8)	2, 8.0 (0.0–18.0)	0 (0.0–0.0)	2, 7.7 (0.0–17.7)
Total	2695 (100.0)	34,782,715 (100.0)		143, 5.3 (4.5–6.2)	268, 9.9 (8.8–11.1)	116, 4.3 (3.6–5.1)	253, 9.4 (8.3–10.5)

Comparison of sex, age group, size of residential area, and education level distributions between the sample in the present study and the total population of Korea. Abbreviations: N, number; CI, confidence interval.

### Prevalence of migraine, self-reported insomnia, anxiety, and depression

Among the 2695 participants, 143 (5.3%) subjects met the ICHD-II criteria for migraine. Insomnia was self-reported in 253 (9.4%) subjects. Anxiety and depression were observed in 268 (9.9%) and 116 (4.3%) participants, respectively (Table 1). The prevalence of self-reported insomnia was significantly higher in migraineurs than in non-migraineurs (21.7% vs. 8.7%. *p* < 0.001). Migraineurs also had a higher prevalence of anxiety (30.1% vs. 8.8%, *p* < 0.001) and depression (16.8% vs. 3.6%, *p* < 0.001) than non-migraineurs.

### Distribution of self-reported insomnia, insomnia symptoms, anxiety, and depression among migraineurs and non-migraineurs

**Figure 2:**
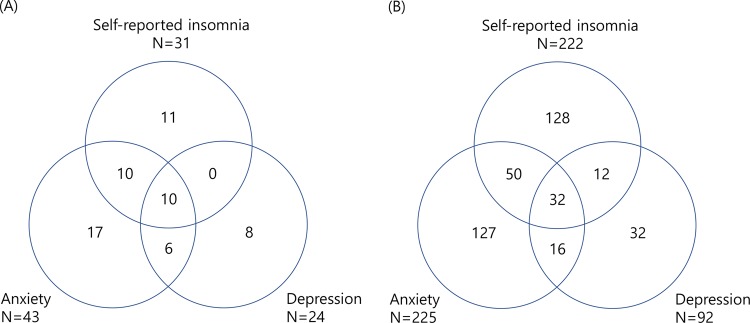
Venn diagrams for the distribution of self-reported insomnia, anxiety, and depression among migraineurs (**A**) and non-migraineurs (**B**).

There was a considerable overlap between self-reported insomnia, anxiety, and depression among migraineurs and non-migraineurs. Among the 143 migraineurs, 31 (21.7%), 43 (30.1%), and 24 (16.8%) participants had self-reported insomnia, anxiety, and depression, respectively. The distribution of self-reported insomnia, anxiety, and depression highly overlapped among migraineurs (Fig. 2A).

Among the 2,552 non-migraineurs, 222 (8.7%), 225 (8.8%), and 92 (3.6%) participants had self-reported insomnia, anxiety, and depression, respectively. The distribution of self-reported insomnia, anxiety, and depression also remarkably overlapped among non-migraineurs (Fig. 2B).

### Sensitivity, specificity, PPV, NPV, and OR for anxiety and depression in all participants with self-reported insomnia

Among the 2,695 participants, the sensitivity and specificity for self-reported insomnia were 38.1% and 93.8% for those with anxiety and 46.6% and 92.3% for those with depression. The PPV and NPV were determined as 40.3% and 93.2% for anxiety and 21.3% and 97.5% for depression. The OR for anxiety and depression was 9.3 (95% CI = 6.9–12.5) and 10.4 (95% CI = 7.0–15.4), respectively.

### Sensitivity, specificity, PPV, NPV, and OR for anxiety and depression in migraineurs with self-reported insomnia

Among the 143 migraineurs, the sensitivity and specificity for self-reported insomnia were 46.5% and 89.0% for those with anxiety and 41.7% and 82.4% for those with depression. The PPV and NPV were determined as 64.5% and 79.5% for anxiety and 32.3% and 87.5% for depression. The OR for anxiety and depression was 7.0 (95% CI = 3.0–16.7) and 3.3 (95% CI = 1.3–8.5), respectively (Tables 2 and 3).

**Table 2:** Sensitivity, specificity, accuracy, positive predictive value, negative predictive value, and odds ratio for anxiety in migraineurs and non-migraineurs with self-reported insomnia. Abbreviations: CI, confidence interval.

	Migraineurs	Non-migraineurs	p-value
Sensitivity, % (95% CI)	46.5 (31.6–61.4)	36.4 (30.2–42.7)	0.233
Specificity, % (95% CI)	89.0 (82.9–95.1)	94.0 (93.0–95.0)	0.054
Accuracy, % (95% CI)	76.2 (69.3–83.2)	88.9 (87.7–90.1)	<0.001
Positive predictive value, % (95% CI)	64.5 (47.7–81.4)	36.9 (30.6–43.3)	0.006
Negative predictive value, % (95% CI)	79.5 (72.0–87.0)	93.9 (92.9–94.8)	<0.001
Odds ratio (95% CI)	7.0 (3.0–16.7)	9.0 (6.5–12.4)	0.608

**Table 3:** Sensitivity, specificity, accuracy, positive predictive value, negative predictive value, and odds ratio for depression in migraineurs and non-migraineurs with self-reported insomnia. Abbreviations: CI, confidence interval.

	Migraineurs	Non-migraineurs	p-value
Sensitivity, % (95% CI)	41.7 (21.9–61.4)	47.8 (37.6–58.0)	0.651
Specificity, % (95% CI)	82.4 (75.5–89.2)	92.8 (91.7–93.8)	<0.001
Accuracy, % (95% CI)	75.5 (68.5–82.6)	91.1 (90.0–92.3)	<0.001
Positive predictive value, % (95% CI)	32.3 (15.8–48.7)	19.8 (14.6–25.1)	0.157
Negative predictive value, % (95% CI)	87.5 (81.4–93.6)	97.9 (97.4–98.5)	<0.001
Odds ratio (95% CI)	3.3 (1.3–8.5)	11.8 (7.6–18.2)	0.017

*p-value: estimated using chi-square test.

### Sensitivity, specificity, PPV, NPV, and OR for anxiety and depression in non-migraineurs with self-reported insomnia

Among the 2,552 non-migraineurs, the sensitivity and specificity of self-reported insomnia were 36.4% and 94.0% for those with anxiety and 47.8% and 92.8% for those with depression. The PPV and NPV were determined as 36.9% and 93.9% for anxiety and 19.8% and 97.9% for depression, respectively. The OR for anxiety and depression was 9.0 (95% CI = 6.5–12.4) and 11.8 (95% CI = 7.6–18.2) (Tables 2 and 3).

Accuracy, PPV, and NPV for anxiety were significantly higher for non-migraineurs than for migraineurs. The sensitivity and specificity for anxiety did not significantly differ between migraineurs and non-migraineurs (Table 2). For depression, the specificity, accuracy, PPV, NPV, and OR were significantly higher among non-migraineurs than among migraineurs. The sensitivity for depression did not significantly differ between migraineurs and non-migraineurs (Table 3).

### Sensitivity, specificity, PPV, NPV, and OR for anxiety and depression in migraineurs with insomnia symptoms

Among the 143 migraineurs, the sensitivity and specificity for insomnia symptoms were 46.5% and 83.0% for those with anxiety and 54.2% and 79.8% for those with depression. The PPV and NPV were determined as 54.1% and 78.3% for anxiety and 35.1% and 89.6% for depression, respectively. The OR for anxiety and depression was 4.3 (95% CI = 1.9–9.4) and 4.7 (95% CI = 1.9–11.7), respectively. The sensitivity, specificity, PPV, NPV, and OR for anxiety and depression based on insomnia symptoms did not significantly differ from those based on self-reported insomnia (Tables 4 and 5).

**Table 4:** Sensitivity, specificity, accuracy, positive predictive value, negative predictive value, and odds ratio of anxiety in migraineurs with self-reported insomnia and those with insomnia symptoms.

	Self-reported insomnia	Insomnia symptom	p-value
Sensitivity, % (95% CI)	46.5 (31.6–61.4)	46.5 (31.6–61.4)	>0.999
Specificity, % (95% CI)	89.0 (82.9–95.1)	83.0 (75.6–90.4)	0.153
Accuracy, % (95% CI)	76.2 (69.3–83.2)	72.0 (64.7–79.4)	0.237
Positive predictive value, % (95% CI)	64.5 (47.7–81.4)	54.1 (38.0–70.1)	0.195
Negative predictive value, % (95% CI)	79.5 (72.0–87.0)	78.3 (70.5–86.2)	0.600
Odds ratio (95% CI)	7.1 (3.0–16.7)	4.3 (1.9–9.4)	0.256

Abbreviations: CI, confidence interval.

*p-value: estimated using generalized estimating equation methods.

**Table 5:** Sensitivity, specificity, accuracy, positive predictive value, negative predictive value, and odds ratio of depression in migraineurs with self-reported insomnia and those with insomnia symptoms.

	Self-reported insomnia	Insomnia symptom	p-value
Sensitivity, % (95% CI)	41.7 (21.9–61.4)	54.2 (34.2–74.1)	0.064
Specificity, % (95% CI)	82.4 (75.5–89.2)	79.8 (72.6–87.0)	0.531
Accuracy, % (95% CI)	75.5 (68.5–82.6)	75.5 (68.5–82.6)	>0.999
Positive predictive value, % (95% CI)	32.3 (15.8–48.7)	35.1 (19.8–50.5)	0.620
Negative predictive value, % (95% CI)	87.5 (81.4–93.6)	89.6 (83.8–95.4)	0.148
Odds ratio (95% CI)	3.3 (1.3–8.5)	4.7 (1.9–11.7)	0.373

Abbreviations: CI, confidence interval.

*p-value: estimated using generalized estimating equation methods.

### Receiver operating characteristics curve of self-reported insomnia and insomnia symptoms for anxiety and depression among migraineurs

**Figure 3:**
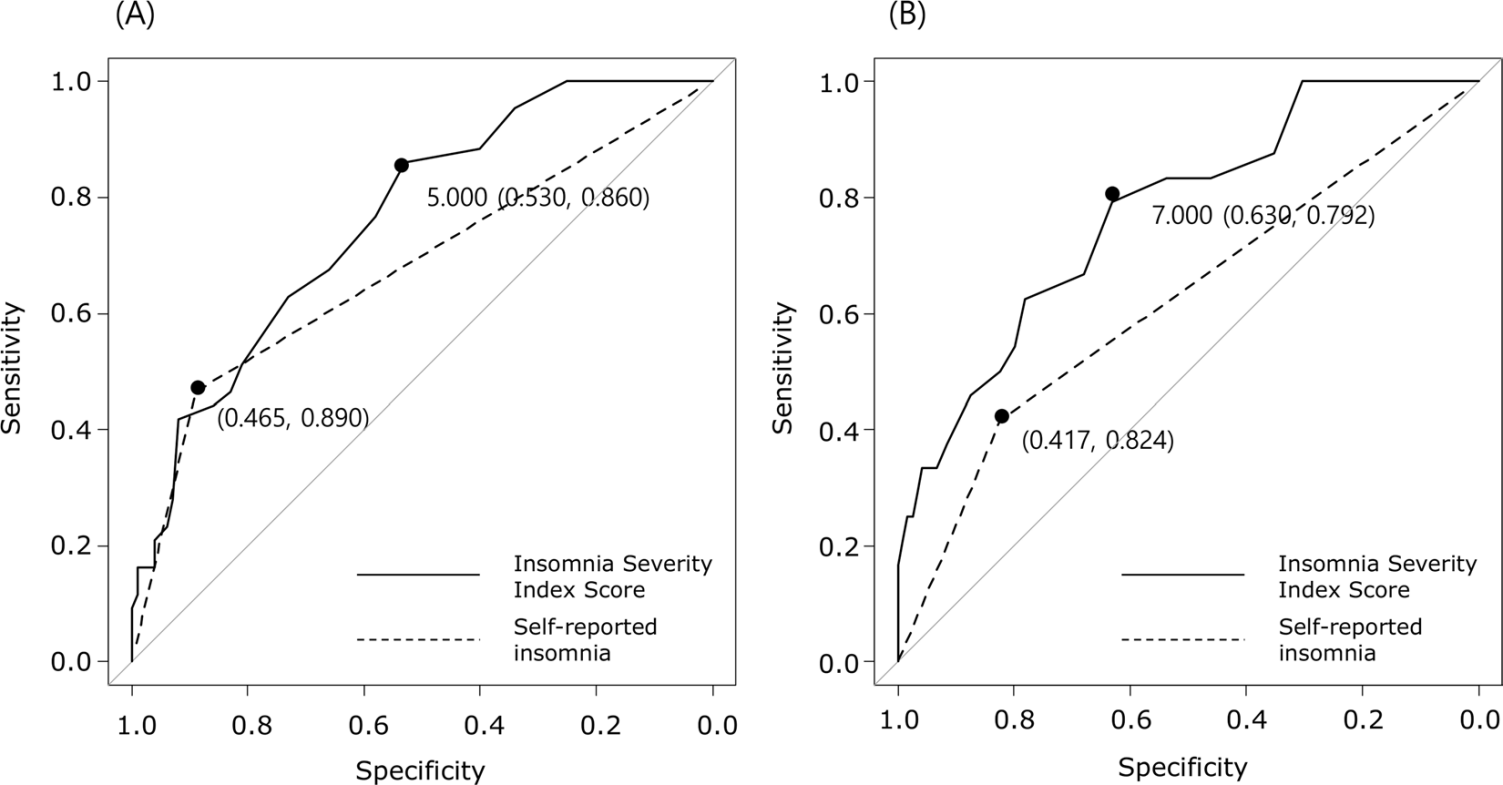
Receiver operating characteristic (ROC) curves of self-reported insomnia (dotted line) and the Insomnia Severity Index score (solid line) for anxiety (**A**) and depression (**B**). The points for the maximal area under the curve are highlighted, with specificity and sensitivity values.

For the evaluation and comparison of diagnostic utility between self-reported insomnia and insomnia symptoms for anxiety and depression, ROCs were generated. The ROC for anxiety achieved the maximal Youden index at an ISI score of 5 with an AUC of 0.762 (95% CI = 0.680–0.843). The AUC of self-reported insomnia was 0.678 (95% CI = 0.571–0.752). The diagnostic utility at an ISI score of 5 was significantly higher than that of self-reported insomnia (*p* = 0.022) (Fig. 3A). The ROC for depression achieved the maximal Youden index at an ISI score of 7 with an AUC of 0.773 (95% CI = 0.671–0.870). The AUC of self-reported insomnia was 0.620 (95% CI = 0.541–0.707). The diagnostic utility at an ISI score of 7 was significantly higher than that of self-reported insomnia (*p* < 0.001) (Fig. 3B).

### Sensitivity, specificity, PPV, NPV, and OR for anxiety and depression in migraineurs with subtypes of insomnia symptoms

We further evaluated the sensitivity, specificity, accuracy, PPV, NPV, and OR according to the subtypes of insomnia symptoms. Sensitivity, specificity, accuracy, PPV, NPV, and OR for anxiety and depression were similar among the different subtypes of insomnia symptoms (see Supplementary Table S1).

## Discussion

We investigated the diagnostic value of self-reported insomnia for anxiety and depression among migraineurs, using data from a population-based sample. We found that self-reported insomnia had high specificity and NPV, and self-reported insomnia could therefore be a useful marker for the exclusion of anxiety and depression among migraineurs.

Insomnia has been reported to have a significant association with migraine. Previous cross-sectional population-based studies have reported a significantly increased risk of insomnia or insomnia symptoms among migraineurs (OR = 1.4 to 2.6)^26,27^. An increased risk of migraine was also noted among individuals with insomnia (OR = 2.4)^28^. In the present study, both an increased risk (21.7% vs. 8.7%, OR = 2.5) of self-reported insomnia among migraineurs and an increased risk of migraine (12.3% vs. 4.6%, OR = 2.7) among participants with self-reported insomnia were noted. The similarity in the ORs for the risk of insomnia among migraineurs and for the risk of migraine among participants with insomnia between the present study and previous studies suggests that both insomnia and migraine were appropriately evaluated.

The present study found a marked co-occurrence of self-reported insomnia, anxiety, and depression among migraineurs—the risk of anxiety and depression increased if self-reported insomnia was present. These findings are consistent with those of previous studies. In a community-based study in the United States, the risk of anxiety (OR = 6.7) and depression (OR = 6.5) among individuals with insomnia was significantly increased compared to individuals without insomnia^29^. Another study conducted in Switzerland showed that individuals with insomnia have an increased risk of anxiety (OR = 8.9) and depression (OR = 15.1) compared to those without insomnia^30^. Our study firstly evaluated the relationship between anxiety, depression, self-reported insomnia, and migraine in a general population-based sample and verified the close association between these conditions.

What are possible mechanisms for the close association among anxiety, depression, self-reported insomnia, and migraine? The most likely explanation is that these conditions share common pathophysiological mechanisms^31^: Bidirectional comorbidity in longitudinal studies suggests a shared mechanism of disorders^32^. Migraine has shown bidirectional comorbidities with insomnia, anxiety, and depression in longitudinal studies^26,33,34^. Significant bidirectional comorbidities of insomnia with anxiety and depression have also been observed^11,35^. Serotonin and dopamine play important roles in the pathogenesis of migraine, insomnia, anxiety, and depression^36,37^, and serotonergic and dopaminergic dysfunctions have been noted in these conditions^38–40^, possibly resulting in the close association between them.

The prevalence of insomnia varies as follows depending on different definitions of insomnia: 1) when the presence of insomnia alone was used as the criterion, the prevalence ranged from 10% to 48%; 2) when insomnia symptoms and daytime consequences were combined, the prevalence was somewhat decreased, ranging between 9% and 15%; 3) when dissatisfaction with sleep quality or quantity was considered, the prevalence ranged from 8% to 18%; and 4) when diagnostic criteria for insomnia were applied, the prevalence was approximately 6%^15^. The present study assessed insomnia by asking a simple question “Do you have insomnia?”, for convenience in actual use. Our insomnia evaluation was based on questions asking about insomnia symptoms or dissatisfaction with sleep quality or sleep quantity, and our group with self-reported insomnia included most participants with criteria- or polysomnography-defined insomnia^41^. The prevalence of self-reported insomnia in the present study was within the range reported in previous studies^15^. Furthermore, the present study found that sensitivity, specificity, PPV, and NPV for anxiety and depression did not significantly differ for participants with self-reported insomnia and those with insomnia symptoms that were assessed using a validated instrument^21^.

The prevalence rates of migraine, anxiety, and depression observed in the present study were similar to those reported in previous studies. The 1-year prevalence of migraine ranges from 4.7% to 9.1% in most studies conducted in Asian countries^42^. We also found prevalence rates of anxiety and depression similar to those previously reported, ranging from 5.6% to 19.3% for anxiety and 3.6% to 6.6% for depression^43–45^.

The estimated sensitivity and PPV for anxiety and depression in participants with self-reported insomnia were not high. In contrast, the specificity and NPV for anxiety and depression in this group were relatively high. These findings are similar to those observed using the nitrite test for urinary tract infections (UTIs). The nitrite test is a fast and cheap method for detecting UTIs. It has a low sensitivity of 37–71% and a high specificity of 82–100.0% and has shown usefulness in excluding UTI^46^. Similarly, self-reported insomnia could be useful in screening or evaluating anxiety and depression. If an individual negatively responds to having self-reported insomnia, the individual has a high probability of not having anxiety and depression. Consequently, self-reported insomnia could be a useful marker in excluding anxiety and depression. Therefore, our findings will be helpful in diagnosing anxiety and depression and will result in more effective treatment of anxiety, depression, and migraine. Further studies in various clinical and population-based settings will be needed to verify our findings on self-reported insomnia as a marker for anxiety and depression.

We used the ISI to assess insomnia symptoms in the present study. The ISI is composed of the following seven items to assess various domains of insomnia symptoms: severity of sleep onset, sleep maintenance, early morning awakening problems, sleep dissatisfaction, interference of sleep difficulties with daytime functioning, noticeability of sleep problems by others, and distress caused by the sleep difficulties. The ISI has been validated for the diagnosis of insomnia, with good sensitivity and specificity, in a population-based setting, by comparison with a doctor’s diagnosis based on the second edition of the International Classification of Sleep Disorders criteria^21^. Several instruments have been used to evaluate sleep in addition to the ISI. The Pittsburg Sleep Quality Index (PSQI) is a validated questionnaire for the investigation of sleep problems. It is composed of 19 items and seven sub-components and has been widely used to evaluate a variety of sleep problems^47^. The PSQI measures a construct (sleep quality) that is related to insomnia; however, sleep quality is broader than just insomnia severity^21^. The Epworth Sleepiness Scale (ESS) is an eight-item instrument that assesses the severity of excessive daytime sleepiness (EDS) in various situations of daily activities. The EDS represents symptoms of broad sleep problems, including poor sleep quality, mood symptoms, sleep-disordered breathing, and metabolic syndrome, in addition to insomnia^48^. The ESS shows a close association with the ISI, but it has not been validated for assessing insomnia in a population-based setting^49^.

The overall response rate in our study was not high. Nevertheless, we adopted a two-stage cluster random sampling method based on the population distribution of Korea. The distributions of age, sex, size of residential area, and education level in our sample did not significantly differ from those in the general population of Korea. Furthermore, the prevalence rates of anxiety, depression, self-reported insomnia, and migraine were similar to those in previous studies^15,42–45^. We can therefore assume an appropriate sampling in the present study.

This study used data from a cross-sectional study. Cross-sectional studies may be at risk for several biases^50^. First, the data only provide a weak evidence on the relationship between exposure and outcomes as it is difficult to separate cause and effect. We did, however, not investigate cause-result associations but evaluated the diagnostic value of self-reported insomnia for identifying anxiety and depression. Second, selection biases occur when study participants differ in their characteristics from eligible participants who were not selected for the study. The sociodemographic distribution of the present study was, however, similar to the sociodemographic distribution of the general population of Korea (Table 1). Third, prior knowledge of the condition might influence the ascertainment of the exposure or the outcome (recall and detection bias). Although our survey was based on participant reports, our structured interviews and questionnaires showed high validity and reliability. The prevalence rates of migraine, self-reported insomnia, anxiety, and depression were similar to those reported in previous studies^41–44^. Fourth, confounding biases can confuse the association between an exposure and an outcome. Migraineurs have higher prevalence rates of self-reported insomnia, anxiety, and depression than non-migraineurs, and this difference may affect the diagnostic value of self-reported insomnia. We found that self-reported insomnia showed a high specificity and a low sensitivity for anxiety and depression both in migraineurs and non-migraineurs, although some diagnostic parameters were significantly different between migraineur and non-migraineurs (Tables 2 and 3). This finding suggests that self-reported insomnia has a diagnostic value regardless of the confounding factor migraine.

Our study has several limitations. First, we assessed insomnia simply by asking our participants “Do you have insomnia?” and did not use objective methods, such as polysomnography or actigraphy. Some patients with sleep-disordered breathing and restless-legs syndrome present with symptoms of chronic insomnia^16^, and although the overall prevalence of insomnia in our study is similar to the results of previous studies, we cannot rule out that other sleep disorders might have been included in our self-reported insomnia group. Second, we did not investigate the use of medication among our participants. Anxiety, depression, and insomnia are common conditions in migraineurs, and antidepressants, anxiolytics, hypnotics, and preventive medications may affect the symptoms of these conditions as well as that of migraine. Lastly, although the present study used data of a population-based study with a large sample size, the lack of significant findings in certain subgroup analyses may have been due to the limited sample size and limited statistical power.

Despite these limitations, the present study has several strengths that should be noted. First, the study has a large sample size and used clustered random sampling proportional to the Korean general population; the estimated sampling error was thus low. Second, we used validated questionnaires for the diagnosis of anxiety, depression, and migraine. The questionnaire showed a high sensitivity and specificity, and the associations found for these conditions are similar to those reported in previous studies. Third, we investigated migraine, anxiety, depression, and insomnia simultaneously, using a large population-based dataset. These conditions have been reported to have close associations, but only few studies have evaluated their relationship in the same dataset. The findings of the present study will be of help in the diagnosis and treatment of migraine, in addition to enhancing our understanding of the relationship between migraine, insomnia, anxiety, and depression. Lastly, we found that self-reported insomnia was a specific marker for anxiety and depression among non-migraineurs as well as migraineurs. Considering that anxiety and depression impose a huge burden globally and the underdiagnosis and undertreatment that are prevalent in these conditions, our findings will greatly help in improving diagnoses and treatments, eventually reducing the burden of anxiety and depression^51^.

## Conclusion

Migraine, self-reported insomnia, anxiety, and depression show a close association in a general population-based sample. Migraineurs with self-reported insomnia have an increased risk of anxiety and depression compared to those without self-reported insomnia. Self-reported insomnia is likely to be comorbid with anxiety and depression among migraineurs and is useful to exclude anxiety and depression.

## Supplementary information

Supplementary Table S1

## Data Availability

Anonymized data relevant to this study will be shared by request with qualified investigator pending appropriate Institutional Review Board approvals.
