# Supplementary material for: Self-reported insomnia as a marker for anxiety and depression among migraineurs: a population-based cross-sectional study
Source: Sci Rep. 2019 Dec 20;9:19608. doi: 10.1038/s41598-019-55928-8 (PMC6925234; doi:10.1038/s41598-019-55928-8)
Supplement: Supplementary file 1 — Supplementary Table S1 [file 41598_2019_55928_MOESM1_ESM.docx]

Supplementary information

Title: Self-reported insomnia as a marker for anxiety and depression among migraineurs: a population-based cross-sectional study

Kyung Min Kim, MD,^1^ Dong Hyun Lee, MD,^1^ Eun Ju Lee, MS,^2^ Yun Ho Roh, MS,^2^ Won-Joo Kim, MD,^3^ Soo-Jin Cho, MD,^4^ Kwang Ik Yang, MD,^5^ Chang-Ho Yun, MD,^6^ and Min Kyung Chu, MD^1*^

^1^ Department of Neurology, Severance Hospital, Yonsei University College of Medicine, Seoul, Korea

^2^ Biostatistics Collaboration Unit, Department of Biomedical Systems Informatics, Yonsei University College of Medicine, Seoul, Korea

^3^ Department of Neurology, Gangnam Severance Hospital, Yonsei University College of Medicine, Seoul, Korea

^4^ Department of Neurology, Dongtan Sacred Heart Hospital, Hallym University College of Medicine, Hwaseong, Korea

^5^ Sleep Disorders Center, Department of Neurology, Soonchunhyang University College of Medicine, Cheonan Hospital, Cheonan, Korea

^6^ Department of Neurology, Bundang Clinical Neuroscience Institute, Seoul National University Bundang Hospital, Seongnam, Korea

|  | **Anxiety** | | | **Depression** | | |
| --- | --- | --- | --- | --- | --- | --- |
|  | **Insomnia symptom-DIS** | **Insomnia symptom-DMS** | **Insomnia symptom-EMA** | **Insomnia symptom-DIS** | **Insomnia symptom-**  **DMS** | **Insomnia symptom -EMA** |
| **Sensitivity, % (95% CI)** | 34.9  (21.0-50.9) | 32.8  (19.1-48.5) | 29.9  (15.3-43.7) | 50.0  (29.1-70.9) | 47.7  (22.1-63.4) | 37.5  (18.8-59.4) |
| **Sensitivity, % (95% CI)** | 91.6  (84.6-96.1) | 91.0  (83.6-95.8) | 92.0  (84.8-96.5) | 89.9  (82.9-94.6) | 89.1  (82.0-94.1) | 90.8  (84.1-95.3) |
| **Sensitivity, % (95% CI)** | 75.3  (67.6-82.0) | 74.4  (65.4-80.5) | 72.7  (64.7-79.8) | 83.2  (75.9-88.9) | 81.1  (73.7-87.1) | 81.8  (74.5-87.8) |
| **PPV, %**  **(95% CI)** | 62.8  (44.1-77.9) | 60.8  (42.2-76.8) | 60.0  (39.8-77.3) | 50.0  (33.9-66.1) | 43.5  (27.7-60.7) | 45.6  (27.6-63.7) |
| **NPV, % (95% CI)** | 77.8  (73.6-81.4) | 75.8  (71.7-79.6) | 74.8  (71.0-78.3) | 89.9  (85.5-93.0) | 88.3  (84.3-91.4) | 87.8  (84.0-90.8) |
| **OR,**  **(95% CI)** | 5.8  (2.3-14.7) | 4.9  (1.9-12.4) | 4.5  (1.7-11.9) | 8.8  (3.3-24.0) | 5.8  (2.2-15.8) | 5.9  (2.1-16.6) |

**Table S1.** Sensitivity, specificity, accuracy, positive predictive value, negative predictive value, and odds ratio of anxiety and depression in migraineurs with subtypes of insomnia symptoms. Abbreviations: CI, confidence interval; DIS, difficulty in initiating sleep; DMS, difficulty in maintaining sleep; EMA, early morning awakening.
